# Transcriptomic Analysis of HCN-2 Cells Suggests Connection among Oxidative Stress, Senescence, and Neuron Death after SARS-CoV-2 Infection

**DOI:** 10.3390/cells10092189

**Published:** 2021-08-25

**Authors:** Andrea Valeri, Luigi Chiricosta, Valeria Calcaterra, Mara Biasin, Gioia Cappelletti, Stephana Carelli, Gian Vincenzo Zuccotti, Placido Bramanti, Gloria Pelizzo, Emanuela Mazzon, Agnese Gugliandolo

**Affiliations:** 1 IRCCS Centro Neurolesi “Bonino-Pulejo”, Via Provinciale Palermo, Contrada Casazza, 98124 Messina, Italy; andrea.valeri@irccsme.it (A.V.); luigi.chiricosta@irccsme.it (L.C.); placido.bramanti@irccsme.it (P.B.); agnese.gugliandolo@irccsme.it (A.G.); 2 Department of Paediatrics, Ospedale dei Bambini “Vittore Buzzi”, 20154 Milano, Italy; valeria.calcaterra@unipv.it (V.C.); gianvincenzo.zuccotti@unimi.it (G.V.Z.); 3 Paediatrics and Adolescentology Unit, Department of Internal Medicine, University of Pavia, 27100 Pavia, Italy; 4 Department of Biomedical and Clinical Sciences-L. Sacco, University of Milan, 20157 Milan, Italy; mara.biasin@unimi.it (M.B.); gioia.cappelletti@unimi.it (G.C.); gloriapelizzo@gmail.com (G.P.); 5 Paediatric Clinical Research Center Fondazione Romeo ed Enrica Invernizzi, University of Milan, 20157 Milan, Italy; stephana.carelli@unimi.it; 6 Paediatric Surgery Unit, Ospedale dei Bambini “Vittore Buzzi”, 20154 Milano, Italy

**Keywords:** oxidative stress, ROS homeostasis, cell cycle, apoptosis, HCN-2, SARS-CoV-2

## Abstract

According to the neurological symptoms of SARS-CoV-2 infection, it is known that the nervous system is influenced by the virus. We used pediatric human cerebral cortical cell line HCN-2 as a neuronal model of SARS-CoV-2 infection, and, through transcriptomic analysis, our aim was to evaluate the effect of SARS-CoV-2 in this type of cells. Transcriptome analyses revealed impairment in *TXN* gene, resulting in deregulation of its antioxidant functions, as well as a decrease in the DNA-repairing mechanism, as indicated by the decrease in *KAT5*. Western blot analyses of SOD1 and iNOS confirmed the impairment of reduction mechanisms and an increase in oxidative stress. Upregulation of *CDKN2A* and a decrease in *CDK4* and *CDK6* point to the blocking of the cell cycle that, according to the deregulation of repairing mechanism, has apoptosis as the outcome. A high level of proapoptotic gene *PMAIP1* is indeed coherent with neuronal death, as also supported by increased levels of caspase 3. The upregulation of cell-cycle-blocking genes and apoptosis suggests a sufferance state of neurons after SARS-CoV-2 infection, followed by their inevitable death, which can explain the neurological symptoms reported. Further analyses are required to deeply explain the mechanisms and find potential treatments to protect neurons from oxidative stress and prevent their death.

## 1. Introduction

Coronavirus is a family of RNA viruses, which infect both mammals and birds. Their one-stranded RNA filament is surrounded by a nucleoprotein, giving the typical appearance of the tubular helix coiled inside the lipid-containing bilayer shell [1]. The name of the family came from membrane observation, where the presence of some clublike spikes recalls a crown [2].

SARS-CoV-2 has the potential to cause life-threatening illness, primarily in the respiratory tract. The European Center of Disease Prevention and Controls states that the five most common symptoms of SARS-CoV-2 are fever, shortness of breath, cough, fatigue/malaise, and confusion [3]. With the pandemic still ongoing, the long-term consequences of the disease are not yet fully elucidated.

The American Center of Disease Control and Prevention defines “Long-COVID” as a list of symptoms that can last weeks or months after first being infected by SARS-CoV-2 or can appear weeks after infection. The so-called “brain-fog”, loss of smell and taste, depression and anxiety, and memory problems are clear indications that the nervous system suffers the consequences of SARS-CoV-2 infection [4,5]. A multisystem inflammatory syndrome in children (MIS-C) has also been described; it represents a new and serious disease that occurs in temporal association with SARS-CoV-2 infection in which neurological involvement can occur [6]. All this evidence indicates that more attention should be paid to the neurologic manifestation of SARS-CoV-2.

The blood–brain barrier (BBB) is a complex association between astrocytes and brain endothelial cells with the aim of preventing access to the central nervous system to all potentially damaging substances and organism, including viruses [7]. SARS-CoV-2 uses its spike proteins, S1, S2, and RBD (particularly S1), to mediate the barrier breakdown [8]. Impairment of the BBB makes all brain cells susceptible to neuroinflammation due to the reaction of the immune system.

It is shown that the immune response to SARS-CoV-2 can be inadequate, meaning that the activation of T cells can be impaired, as well as the number of B and NK cells; this immune dysregulation favors the expression of genes involved in inflammation and oxidative stress, and cytokine storm can be the next step [9]. Cytokine storm is responsible for multiple organ failure, coma, and death in severe cases of SARS-CoV-2 infection [10,11], but is not well understood as the effect of oxidative stress in the brain of COVID-19 patients.

Oxidative stress is involved in a wide variety of neurodegenerative disorders in adults, such as Alzheimer’s and Parkinson’s diseases, as well as neuropsychiatric disorders [12] and neurological disease in pediatrics [13]. Reactive oxygen species (ROS), the main contributors to oxidative stress, are a natural byproduct of oxidative phosphorylation from mitochondria [14]. Under normal conditions, the ROS level is under control of the antioxidant system, preventing the oxidation of proteins and the peroxidation of lipids. Oxidative stress occurs when the amount of ROS is no longer under the control of the antioxidant system. During oxidative stress, there is the generation of highly reactive species, including singlet oxygen, superoxide anion, hydrogen peroxide, hydroxyl radical, and peroxyl radical [15], which results in DNA, protein, lipid damage [16,17], and, ultimately, cell death. Oxidative stress compromises the permeability of BBB, followed by neuroinflammation and neuronal death [12]. Oxidative stress can damage the different types of cells involved in the architecture of BBB, and, in particular, the damage to tight junctions and basement membrane proteins is involved in BBB leakage. Tight junctions block water-soluble molecules, as well as ions, drugs, and pathogens; thus, their disruption represents a critical loss of BBB integrity; endothelial cells associate with pericytes and astrocytes to mediate the formation of the tight junction [18]. BBB breakdown disrupts its protective properties, because leakage permits entry into the brain of blood products, other cells, and pathogens, which can trigger the response of the immune system. The brain interstitial fluid composition allows the correct functioning of the synapses; hence, an alteration due to excessive permeability can impair the neuronal connectivity, along with signal transmission. Thus, it is not surprising to find an association between BBB breakdown and the most common neurodegenerative diseases [19]. Indeed, oxidative damage to BBB has a main role in neurological disorders such as stroke and Alzheimer’s disease. In Alzheimer’s disease, beta-amyloid produces ROS by damaging the BBB, in turn producing more ROS, which enhances secretase activity and promotes the generation of more beta-amyloid [20]. In order to better understand the BBB breakdown and its connection with viral infection, a mouse model of viral encephalitis using T3A virus was made. Postmortem analyses of pup brains revealed increased cell death and vascular hyperplasia; most importantly, intraperitoneal injection of the virus also caused BBB leakage [21].

In our work, we chose to use pediatric HCN-2 human cortical neurons [22,23] to identify the mechanism of SARS-CoV-2 infection without interaction with other cell types. Cultured neurons were incubated with SARS-CoV-2 and, following observation of actual infection, RNA was collected for transcriptome analysis. The aim of this study was to investigate pathways that are influenced by SARS-CoV-2 infection using next-generation sequencing analysis.

## 2. Materials and Methods

### 2.1. Virus

After the expansion of SARS-CoV-2 (Virus Human 2019-nCoV strain 2019-nCoV/Italy-INMI1, Rome, Italy) on Calu-3 cells (ATCC^®^ HTB-55™), we calculated TCID_50_ as previously reported [24]. All experiments with SARS-CoV-2 were conducted at a BSL3 facility.

### 2.2. In Vitro HCN-2 SARS-CoV-2 Infection Assay

HCN-2 cells were provided by ATCC (CTRL-10742) and were cultured in DMEM (Euroclone, Milan, Italy) + 10% FBS; 100 U/mL penicillin and 100 μg/mL streptomycin were used as antibiotics. The cells were cultured in a 25 cm^2^ culture flask. The same medium, without FBS, was used as inoculum in the mock-infected cells. Cell cultures were incubated with 1 multiplicity of infection (MOI); the incubator was set at 37 °C and 5% CO_2_ for 3 h, after which cells were washed two times with lukewarm PBS and refilled with the growth medium (+ 10% FBS). In order to assess the cytopathic effect, we checked the cells daily using an optical microscope (ZOE™ Fluorescent Cell Imager, Bio-Rad, Hercules, CA, USA). RNA was extracted from mock and infected cells. The protocol used was previously described [25]. Using single-step real-time PCR (GoTaq^®^ 1-Step RT-qPCR) (Promega, Fitchburg, WI, USA), viral RNA was quantified on a CFX96 (Bio-Rad, Hercules, CA, USA) using primers against two regions of the nucleocapsid (N1 and N2) gene of SARS-CoV-2 (2019-nCoV CDC qPCR Probe Assay emergency kit; IDT, Coralville, IA, USA). The standard curve was generated after quantification of 2019-nCoV_N Positive Plasmid Control (IDT, Coralville, IA, USA). All procedures were performed in agreement with the GLP guidelines adopted in our laboratory.

### 2.3. RNA-Seq Analysis

The library preparation was carried out according to the TruSeq RNA Exome protocol (Illumina, San Diego, CA, USA) following the instructions. The libraries were sequenced with the Illumina MiSeq Instrument, and the raw data check was performed using the fastQC tool. Taking advantage of Trimmomatic (version 0.38, Usadel Lab, Aachen, Germany) [26], we trimmed adapters and bases with low quality. The human reference genome GRCh38 was used to align the reads using the Spliced Transcripts Alignment to a Reference (STAR) RNA-seq aligner [27], and the counting was conducted through the python package htseq-count [28]. The package DESeq2 of Bioconductor [29] was used on R to analyze the differentially expressed genes. All genes with a *q*-value lower than 0.05 after the post hoc Benjamini–Hochberg procedure were kept in the downstream analysis. No fold-change thresholds were used. Differentially expressed genes (DEGs) were then enriched with Gene Ontology, particularly the Biological Process category, using the cluster Profiler library [30]. The terms with a *q*-value higher than 0.05 were rejected. Lastly, the pathways in which DEGs were included were inspected using Reactome [31].

### 2.4. Culture Medium Western Blot

In order to quantify the proteins in the culture medium, cold acetone was added to the culture medium in a proportion of 4:1 and incubated at −20 °C. After 1 h, the tube was centrifuged at 14,000× *g* for 12 min at 4 °C. The supernatant was discarded, and the pellet was resuspended in RIPA buffer. Bradford Assay (Bio-Rad, Hercules, CA, USA) was used to determine the protein concentration. After quantification, an equal amount of protein from control and from infected culture medium was subject to SDS-PAGE and blotted on polyvinylidene fluoride membranes (PVDF) (Immobilon-P transfer membrane; Millipore, Burlington, MA, USA). Ponceau S stain was used to confirm that the proteins were loaded in an equal amount. Blocking of nonspecific binding was performed by incubating the membranes with TBS + 5% skimmed milk for 1 h at room temperature. Primary antibodies used for overnight incubation at 4 °C were anti-superoxide dismutase 1 (1:1000; Abcam, Cambridge, UK; ab16831), anti-caspase 3 (1:1000, Cell Signaling, Danvers, MA, USA; #9662), anti-cyclooxygenase 2 (1:500; Santa Cruz Biotechnology, Dallas, TX, USA; sc-166475), and anti-nitric oxide synthase (1:500; Santa Cruz Biotechnology, Dallas, TX, USA; sc-651). The secondary antibody used for 1 h room temperature incubation was mouse anti-rabbit IgG-HRP (1:2000; Santa Cruz Biotechnology, sc-2357) or chicken anti-mouse IgG (1:2000; ThermoFisher Scientific, Waltham, MA, USA; cat. num. SA1-72021). An ECL system (Luminata Western HRP Substrates; Millipore) was used to visualize protein bands; then, acquisition was done using ChemiDoc MP System (Bio-Rad, Hercules, CA, USA), and ImageJ (National Institute of Health) was used to quantify them.

### 2.5. Statistical Analysis

GraphPad Prism 6.0 (GraphPad Software, La Jolla, CA, USA) was used for statistical analysis. We used Student’s *t*-test to compare the two groups. A *p*-value less than 0.05 was considered statistically significant.

## 3. Results

### 3.1. Virus Replication

At day 1, day 3, and day 6, the replication of the virus was checked by analyzing N1 and N2. As shown in Figure S1, from day 1, we observed an increase n both N1 and N2 values, indicating an efficient replication of the virus. The replication rate increased over time, as is clear from N1 and N2 copy numbers at day 3 and day 6, when the cells were harvested for further analyses.

### 3.2. Enrichment Analyses

**Figure 1:**
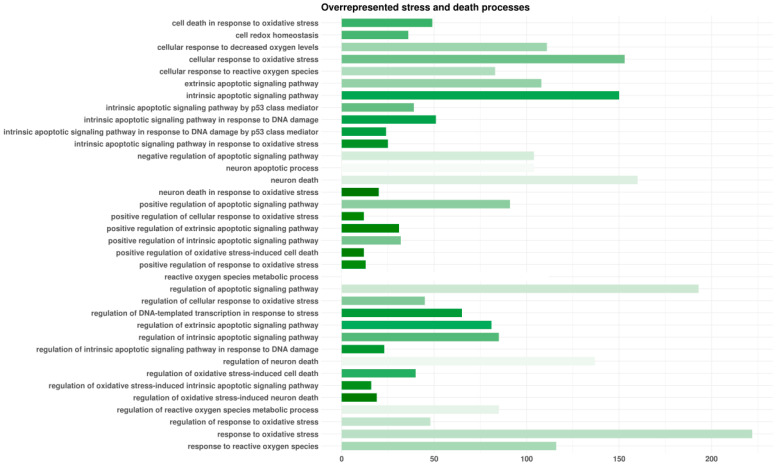
Enriched biological processes terms involved in oxidative stress and death by differentially expressed genes (DEGs) found in HCN-2 cells after exposure to SARS-CoV-2. For each Gene Ontology term represented, the length of the bar shows the number of DEGs observed in that specific category. The color of the bar points to the ratio between the number of DEGs found in our analysis and the number of genes included in the term from white (no DEGs of the term) to green (all DEGs of the term). The terms are alphabetically sorted.

**Figure 2:**
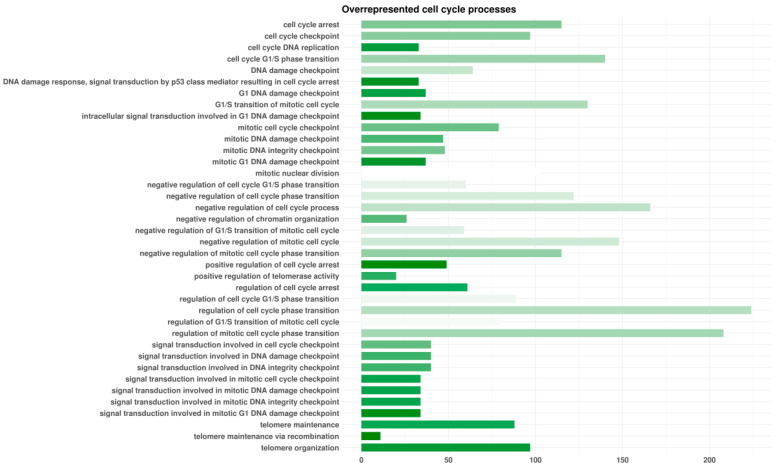
Enriched biological processes terms involved in cell cycle by differentially expressed genes (DEGs) found in HCN-2 cells after exposure to SARS-CoV-2. For each Gene Ontology term represented, the length of the bar shows the number of DEGs observed in that specific category. The color of the bar points to the ratio between the number of DEGs found in our analysis and the number of genes included in the term from white (no DEGs of the term) to green (all DEGs of the term). The terms are alphabetically sorted.

HCN2 showed 7315 genes that differed in a significant manner after the RNA-seq analysis of HCN2-SARS-CoV-2 with respect to HCN2-CTR. Among them, 3527 were upregulated and 3788 were downregulated DEGs. We enriched DEGs with the Gene Ontology domain “Biological Process”. Among the overrepresented terms, we identified 36 classes related to oxidative stress and cell or neuron death (Figure 1). Furthermore, we observed 38 terms associated with cell cycle (Figure 2).

### 3.3. Pathway Inspection

We examined in depth the genes that were down- or upregulated after the SARS-CoV-2 infection that were included in the Reactome “oxidative stress-induced senescence” and “DNA damage/telomere stress-induced senescence” pathways. Moreover, we included in the table the genes implicated in inflammation activation, production of ROS, and apoptosis trigger under ROS persistence. Thus, in Table 1, we included the genes whose fold-change deregulation was at least 0.5. In detail, *FOS* and *CCNE2* genes had a downregulation higher than twofold. *HMGA2*, upregulated, and *CXCL8* and *NOXA1*, downregulated, had a fold-change ranging from 1–2. *CDKN2A, EZH2, MAP3K5, MOV10, ASF1A, CCNA2, CCNE1, HIRA*, and *HMGA1* had a fold-change ranging from 0.5–1. Additionally, in Table S1, we included *AGO1, AGO3, CDK4, CDK6, MAP4K4, MAPKAPK2, MAPKAPK3, MDM4, MINK1, RPS27A, TNRC6A, TNRC6C, TXN, UBA52, UBB, UBC, ATM, CABIN1, CDK2, CDKN1B, EP400, KAT5, MRE11*, and *SOD1*, which still were deregulated in a statistically significant manner but with a fold-change lower than 0.5.

### 3.4. Western Blot Analyses

**Figure 3:**
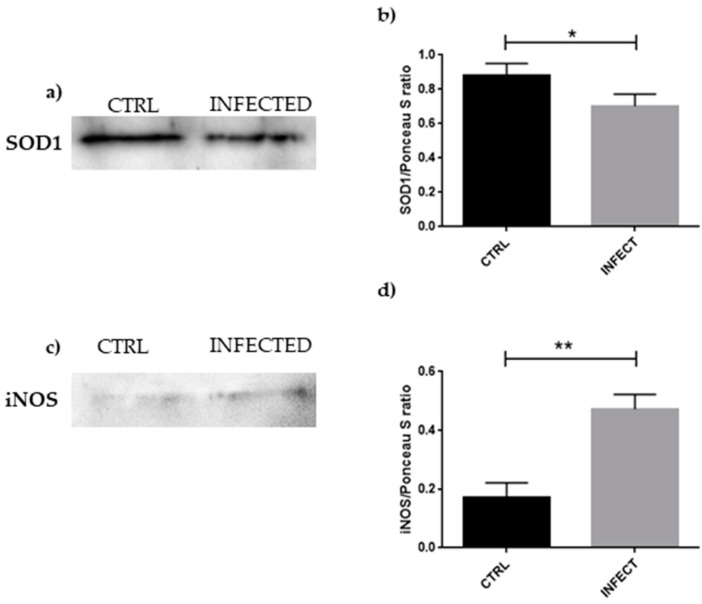
(**a**) Evidence of a significant decrease in *SOD1* expression in HCN-2 culture medium after SARS-CoV-2 infection, suggesting impairment of ROS reduction. (**b**) Densitometric analysis of SOD1, * *p* < 0.05. (**c**) Evidence of significant increase in iNOS expression in HCN-2 culture medium after SARS-CoV-2 infection, as an indicator of reactive species production. (**d**) Densitometric analysis of iNOS, ** *p* < 0.01.

To confirm oxidative stress hypothesized from the RNA-seq analysis, we performed a Western blot investigation of SOD1 and iNOS, the results of which are presented in Figure 3.

Western blot and statistical analyses showed an increase in iNOS levels in parallel with a reduction in SOD1 amount in SARS-CoV-2-infected HCN-2 cells, suggesting the presence of oxidative stress and the impairment of antioxidant mechanisms.

**Figure 4:**
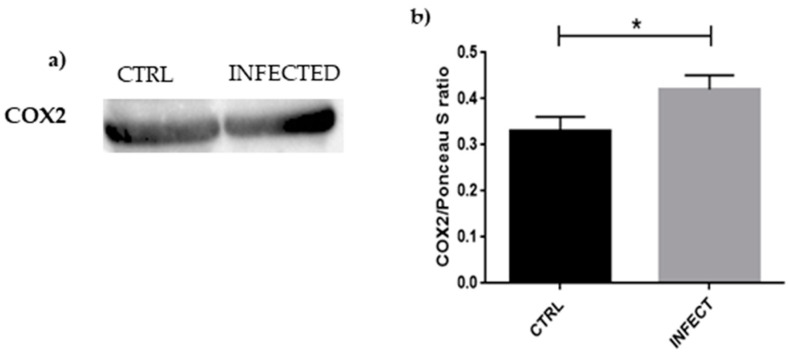
(**a**) Evidence of significant increase in COX2 expression in HCN-2 culture medium after SARS-CoV-2 infection, suggesting inflammation in the infected cells; (**b**) densitometric analysis of COX2, * *p* < 0.05.

Western blot and statistical analyses, shown in Figure 4, also evidenced a significant increase in COX2 levels in HCN-2 cells after SARS-CoV-2 infection, confirming the presence of inflammation.

**Figure 5:**
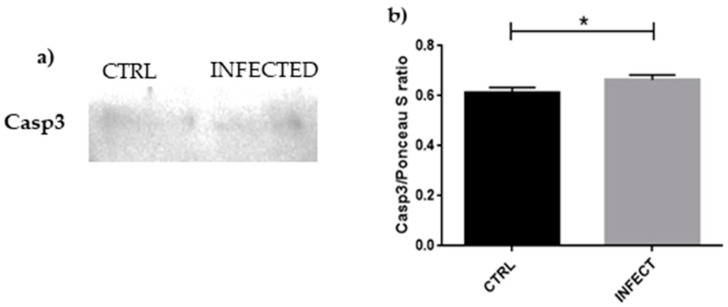
(**a**) Evidence of significant increase in caspase 3 expression in HCN-2 culture medium after SARS-CoV-2 infection, suggesting that apoptosis is taking place in the infected cells; (**b**) densitometric analysis of caspase 3, * *p* < 0.05.

To confirm the apoptosis of HCN-2 following SARS-CoV-2 infection, we checked the expression of caspase 3. As shown in Figure 5, the level of caspase 3 increased in HCN-2 after SARS-CoV-2 infection.

## 4. Discussion

The neurological effects of SARS-CoV-2 infection suggest that the response of neurons in the presence of the virus should be evaluated. HCN-2 is a human pediatric cortical neuron culture. Free from any other kind of cells, such as microglia, astrocytes, and epithelial cells, the effect of the infection on neurons can be evaluated without any external interference. Using electronic databases of articles based on pediatric outcomes of SARS-CoV-2 infection, it becomes clear that, even if neurological complications are not really frequent in children, those with pre-existent severe illness are at more risk of developing seizures and encephalitis after COVID-19 [32]. This indicates SARS-CoV-2’s potential ability to invade the nervous system.

Our results evidenced an increase in N1 and N2 copy numbers, suggesting that SARS-CoV-2 succeeded in penetrating the cells and replicating itself normally. In order to understand how SARS-CoV-2 may influence the neurons, we used the Biological Process domain of the Gene Ontology dictionary. Our genes were involved in a high number of terms, but the majority of them can be clustered as stress and death processes, as shown in Figure 1. Going more in depth in the analyses, “regulation of apoptotic signal pathway”, “response to oxidative stress”, and “neuron death” were the three where a large quantity of our genes was involved. We noticed also that our genes covered a relevant number of steps in “neuron death in response of oxidative stress” and “regulation of oxidative stress-induced neuron death” processes.

**Figure 6:**
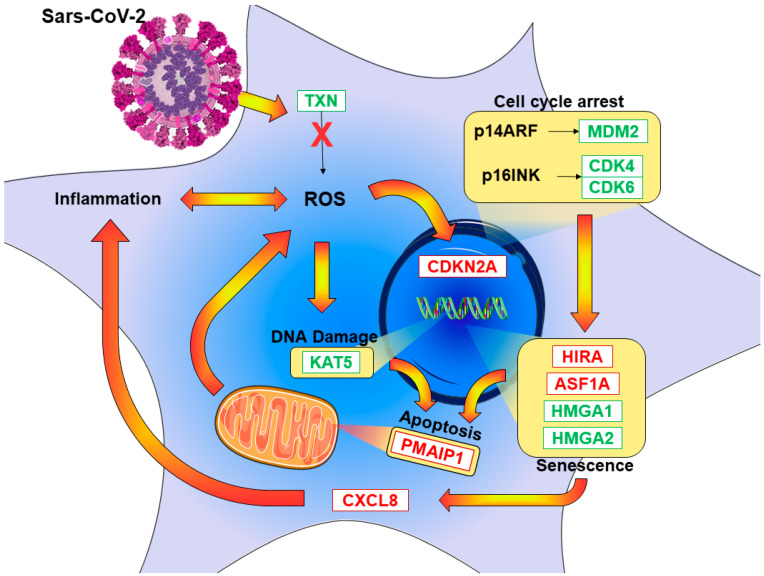
SARS-CoV-2-triggered pathway depiction of DEGs found in RNA-seq analyses after infection of the HCN-2. The red genes are upregulated and, thus, more expressed after the infection. The green downregulated genes are more expressed in the control. The yellow frames show increased activities.

Furthermore, cell-cycle processes contained a relevant number of our genes, as explained in Figure 2. Among them, our genes covered a significant part of “telomere maintenance”, “positive regulation of cell arrest”, and “signal transduction involved in mitotic G1 DNA damage checkpoint”. Using Reactome, we investigated the oxidative stress pathway and senescence as a consequence of the blocking of the cell cycle. Figure 6 summarizes the pathways that we found influenced by SARS-CoV-2, along with the main genes involved in the pathways.

A correlation exists between oxidative stress and viral infection [33]; hence, we first investigated how the cell may defend itself against oxidative stress following SARS-CoV-2 entry. We found that the expressions of *TXN* and *SOD1* were reduced. *TXN* encodes Thioredoxin and catalyzes a bimolecular nucleophilic substitution reaction (S_N_2); the disulfide bonds from the protein targeted by Thioredoxin are transferred to the Thioredoxin itself, reducing the protein target and stabilizing Thioredoxin through oxidation [34]. Since SARS-CoV-2 contains cysteine-rich spike glycoproteins, an infection mechanism involving Thioredoxin was proposed, where the surface proteins can benefit from an ROS-rich environment [35]. Inhibition of Thioredoxin activity causes severe impairment of all reductive capacity of the cell, accumulation of ROS, and a persistent oxidized condition of all Thioredoxin substrates [36], ultimately resulting in apoptosis [37]. SOD1 is an enzyme, whose action consists of converting superoxide radicals to hydrogen peroxide and then water following further reduction [38]. After infection, we found a reduction in *SOD1* expression in neurons, which is supported by protein quantification, as is clear in Figure 3a,b. The role of SOD1 in SARS-CoV-2 infection was not fully elucidated, but H5N1 viral infection in A549 cells revealed increased ROS levels and downregulation of antioxidant enzymes, including SOD1. Knocking out *SOD1* resulted in an increase in viral replication [39]. SARS-CoV-2 may follow a similar mechanism, altering the antioxidant defense of the cell to create a more favorable environment for its replication. SOD1 represents the “first-line defense of antioxidants”, while Thioredoxin is categorized as the “second-line defense”; their decreased level suggests that defense against oxidative stress is impaired at different levels [40], resulting in increased ROS in HCN-2 cells because of incomplete or insufficient reduction capacity. ROS are also increased by *NOXA1*, found overexpressed in our study, as shown in EAhy926 cells, where the production of ROS was decreased after knockdown of *NOXA1* by siRNA [41]. *EP400*, encoding p400, downregulated in our experiments, also has a role in ROS formation. In U2OS osteosarcoma cells, the lack of p400 increased ROS level, leading to DNA damage [42]. As confirmation of the increased oxidative stress, the level of iNOS was shown to be higher in HCN-2 cells after SARS-CoV-2 infection. iNOS is inducible nitric oxide synthase and is responsible for the conversion of l-arginine to l-citrulline, a process that generates nitric oxide. In the presence of oxygen, nitric oxide can be rapidly oxidized to nitrite and nitrate. Reactive nitrogen species contribute then to the increase in oxidative stress. They are also known to correlate with aging, particularly age-related inflammation, as demonstrated by increased nitric oxide metabolite and iNOS amount in aged rats compared to young ones [43]. There is a connection between neurodegenerative diseases and ROS, and iNOS inhibition appears to protect the brain from oxidative stress damage [44].

An excessive level of ROS in the cell leads to DNA damage, as indicated by the upregulation of the *ATM* gene [45]. It was reported that *ATM* can be activated by ROS [46].

Once the DNA is damaged, the cellular repair mechanism will attempt to correct it, in order to avoid apoptosis or transmission of incorrect genetic information. *KAT5*, the gene that encodes the enzyme histone acetyltransferase *KAT5* (Tip60), was downregulated. Mutation of Tip60 in cells resulted in deficiency of DNA reparation, but cells were resistant to apoptosis, indicating a secondary role of Tip60 in transmitting DNA damage to the apoptotic system [47].

The normal progression of the cell cycle also seems to be impaired, in part due to the DNA damage checkpoints, but we can also recognize a role of ROS in cell-cycle arrest. *CDKN2A*, upregulated in our experiment, encodes two proteins, tumor suppressor 14ARF and senescence marker p16INK4A [48]. P14ARF can inhibit cell-cycle progression via p53. P53 is complexed with its inhibitor *MDM2*, but this binding can be weakened by p14ARF, as shown in a cell culture experiment with p19ARF, the mouse homolog of the human p14ARF, where the capacity of p19ARF to inhibit *MDM2*-mediated ubiquitination of p53 is reported [49]. In our study, *MDM2* and *MDM4*, equal in function and similar in structure, were downregulated, suggesting that p14ARF is free to interact with p53 to block the progression of the cell cycle. However, p14ARF does not need p53 for inducing apoptosis, as shown in p53-deficient cells, where p14ARF was able to block cell progression at the G2 stage and trigger apoptosis, if DNA damage was not corrected [50]. Overexpression of *MAPAPK3* induces dissociation between chromatin and Bmi1, a repressor of the *CDKN2A* gene, and results in re-expression of p14ARF protein [51]. P16INK4A, the second protein encoded by the *CDKN2A* gene, interacts with *CDK4* and *CDK6,* both decreased in our study, reinforcing the blocking of the cell cycle [52].

P16INK4A is also a senescence marker. Senescence is a cellular condition where the cell cycle is blocked; thus, there is permanent proliferation arrest [53], but the cell is not completely frozen. Even if the cell cycle is blocked, it is possible for the cell to re-enter the normal cell cycle flow, but this does not seem to be the case in SARS-CoV-2 infection. *CDK2* levels and its interacting Cyclins were downregulated. *CCNA2* encodes CyclinA2, and loss of function of both *CDK2* and CyclinA2 in conditional knockout mice resulted in a slowdown of cell proliferation and premature senescence [54]. *CCNE1* encodes CyclinE1 and *CCNE2* encodes CyclinE2. Studies revealed that these types of Cyclins are not necessary to stop the cell cycle, but cells cannot re-enter in the cell cycle without them [55].

The increased value of *ASF1A* and *HIRA*, which are part of the developing process of senescence-associated heterochromatin foci, reinforces the cell-cycle block and confirms the senescent status of the cells [56]. Furthermore, HMGAs, encoding for the chromatin proteins responsible for changing its architecture, were found to be overexpressed. HMGAs are normally located in the nucleus, but they move in heterochromatin foci associated with senescence when the cells stop growing. Overexpression of *HMGA1* strongly stops the cell cycle [57].

The cell, even if senescent, is still metabolically active; in its secretome, proinflammatory cytokines are found such as IL-8, which can contribute to reinforcing the production of ROS. Inflammation and cell metabolism are connected, and mitochondria production of ROS can be influenced by inflammation-inducing stimuli [58]. IL-8 production induced by SARS-CoV-2 was clear in Calu-3 cells, along with other inflammatory cytokines [59]. IL-8, encoded by the *CXCL8* gene, was proposed to be a better biomarker of the COVID-19 outcome than IL-6, as IL-6 may activate the pro- and anti-inflammatory pathways. IL-8, on the other hand, is clearly proinflammatory [60]. This is consistent with our evidence of an increased value of *CXCL8*. As confirmation of inflammation, *PTGS2* level was increased and its protein COX2, as s reported in Figure 4, resulted upregulated following viral entry. COX2 mediates the synthesis of prostaglandins, which play a role in inflammation, such as increasing vascular permeability that allows the activity of proinflammatory cells, proteins, and enzymes [61]. In an experiment with fibroblasts, COX2 level resulted increased after H_2_O_2_ cell treatment, suggesting a connection among COX2, inflammation, and oxidative stress [62]. Interestingly, in Calu-3 and A549 cells, as well as in the lung of human ACE2-expressing mice, SARS-CoV-2 was able to enter and increase the expression of *PTGS2*. To prove the role of COX2 in the inflammatory response after SARS-CoV-2 infection, the mice were treated with NSAIDs and, even if there was no effect on viral entry and replication, a decrease in cytokine production was observed [63]. COX2 is also upregulated during senescence, as was demonstrated by a comparison between COX2 levels of young human dermal and prostatic fibroblasts and senescent ones. Inhibition of COX2 activity resulted in a decrease in senescence markers. Moreover, COX2 inhibitors were proven to ameliorate the cognitive ability of aging rats [64], while its level is significantly increased in the kidney of 20 month old rats compared to 6 month old ones [43].

The senescent status gives to the cell higher resistance to apoptosis [53]. *MAPK3K5* encodes for ASK-1 protein, which was diminished in value in our experiment. ASK-1 is complexed by Thioredoxin [65], and this binding prevents apoptosis, as reported in an in vitro model of Parkinson’s disease, where Thioredoxin shows inhibition of the proapoptotic protein ASK-1, and where compounds that enhance this binding act as cell protectors [66]. *JUN* and *FOS* were reduced. During an experiment where these gene activities were neutralized via antibody microinjection, neurons were protected against apoptosis mediated by NGF deprivation [67], showing that *JUN* and *FOS* are important for the apoptosis process. *FOS*, in particular, seems strongly downregulated, and its role in neuron survival was investigated; mice with knockout of c-fos in hippocampal neurons had more neuronal loss compared to wild-type ones after kainic acid-induced seizures. The role of c-fos in neuronal protection was elucidated after excessive stimulation, as part of the AP-1 complex along with c-jun, since AP-1 can help cell survival via BDNF regulation [68]. Moreover, *FOS* seems to be a reporter of poor neuronal activity in rats, and we can speculate that its low value reflects the low activity of our cultured neurons [69]. Taken together, these results suggest that the apoptosis pathway may be initially impaired, but there is also the indication that neurons are not very active, and their survivability is not sustained.

The persistent blocking of the cell cycle and the oxidative stress lead to DNA damage that cannot be repaired, promoting apoptosis. This is suggested by the increased level of the proapoptotic gene *PMAIP1*, part of the p53 apoptotic cascade, along with the mechanism of activation of *ATM* in the presence of the ROS mentioned above, suggesting that the cellular death pathway is activated instead of the damage repair pathway, where ROS are the triggers of apoptosis. Apoptosis is confirmed by the increased level of caspase 3. In particular, the oxidative stress confirmed by iNOS increase cannot be counteracted, as demonstrated by the reduced levels of SOD1, leading to apoptosis. An experiment with primary culture of rats showed a connection between iNOS and caspase 3, because inhibition of nitric oxide synthesis seems to reduce caspase 3-like activity [70]. Using SH-SY5Y neuroblastoma cells to evaluate neuronal apoptosis, caspase 3-like activity was activated during apoptosis after NOC18 treatment, a nitric oxide donor, and suppressed after oxyhemoglobin administration, which functioned as a nitric oxide trapper [71].

As confirmation of neuronal death, connecting SARS-CoV-2 infection and neurological impairment, the *EZH2* gene was found to be downregulated. This is associated with loss of neurons and an impairment of spatial learning and memory in mice. Knockout of the *EZH2* gene in the central nervous system leads to impairment in the normal growth of pups, and inducible knockout of *EZH2* in adult mice results in severe impairment of the learning process, spatial memory, contextual fear memory, and pattern separation [72]. Neurons that overexpress *AGO1*, as found in our experiment, have decreased dendritic spine density, if mature, and less complexity, if immature [73].

## 5. Conclusions

The transcriptomic analysis of HCN-2 after SARS-CoV-2 infection indicated inflammation and impairment of the antioxidant defense mechanisms of the cell, leading to increased ROS presence in the neurons. DNA damage, as a consequence of ROS activity, cannot be efficiently corrected, and the cell cycle is blocked, leading to senescence. This status, along with ROS continuous accumulation, triggers an inflammatory response that reinforces the production of ROS until apoptosis is triggered, and this can explain the neurological consequences of SARS-CoV-2 infection. Further experiments are required to deeply investigate SARS-CoV-2 infection mechanisms in neurons; given the deleterious effects of ROS, future antioxidants treatment could be tested to prevent neuronal damage in COVID-19.

## Figures and Tables

**Table 1:** Inspected DEGs involved in stress.

Gene	HCN2-CTR Expression	HCN2-SARS-CoV-2 Expression	Fold Change	*q*-Value	Biological Process
*CDKN2A*	53.60	86.25	0.69	3.10 × 10^−4^	Oxidative stress Induced senescence
*EZH2*	96.00	53.75	−0.84	1.73 × 10^−5^	Oxidative stress Induced senescence
*FOS*	172.00	11.25	−3.93	1.75 × 10^−28^	Oxidative stress Induced senescence
*JUN*	413.61	209.99	−0.98	6.81 × 10^−26^	Oxidative stress Induced senescence
*MAP3K5*	50.40	35.00	−0.53	4.48 × 10^−2^	Oxidative stress Induced senescence
*MOV10*	493.61	324.99	−0.60	1.42 × 10^−14^	Oxidative stress Induced senescence
*ASF1A*	46.40	67.50	0.54	1.18 × 10^−2^	DNA damage/telomere stress-induced senescence
*CCNA2*	92.00	53.75	−0.78	8.33 × 10^−5^	DNA damage/telomere stress-induced senescence
*CCNE1*	82.40	53.75	−0.62	2.50 × 10^−3^	DNA damage/telomere stress-induced senescence
*CCNE2*	40.80	5.00	−3.03	6.73 ×10^−8^	DNA damage/telomere stress-induced senescence
*HIRA*	11.20	21.25	0.92	2.54 × 10^−2^	DNA damage/telomere stress-induced senescence
*HMGA1*	5586.56	3476.15	−0.68	1.20 × 10^−194^	DNA damage/telomere stress-induced senescence
*HMGA2*	1301.64	338.74	−1.94	4.68 × 10^−183^	DNA damage/telomere stress-induced senescence
*CXCL8*	25.60	73.75	1.53	7.78 × 10^−11^	Oxidative stress Alteration
*NOXA1*	3.20	8.75	1.45	4.49 × 10^−2^	Oxidative stress Alteration
*PMAIP1*	23.20	37.50	0.69	1.97 × 10^−2^	Oxidative stress Alteration
*PTGS2*	21.6	343.74	3.99	3.73 × 10^−82^	Oxidative stress Alteration

The fold-change columns are based on log_2_(HCN2-CTR expression/HCN2-SARS-CoV-2 expression). The values are rounded to the second decimal digit.

## Data Availability

The data presented in this study are openly available in the NCBI Sequence Read Archive at BioProject accession number PRJNA742373.

## Supplementary Materials

The following are available online at https://www.mdpi.com/article/10.3390/cells10092189/s1: Figure S1. Replication of SARS-CoV-2 in cultured cells; Table S1. Inspected DEGs involved in stress with fold change <0.5.
