# Supplementary material for: Transcriptomic Analysis of HCN-2 Cells Suggests Connection among Oxidative Stress, Senescence, and Neuron Death after SARS-CoV-2 Infection
Source: Cells. 2021 Aug 25;10(9):2189. doi: 10.3390/cells10092189 (PMC8472605; doi:10.3390/cells10092189)
Supplement: Supplementary file 1 [file cells-10-02189-s001.zip › cells-1332292-supplementary.pdf]

cells
MDPI

Supplementary Materials

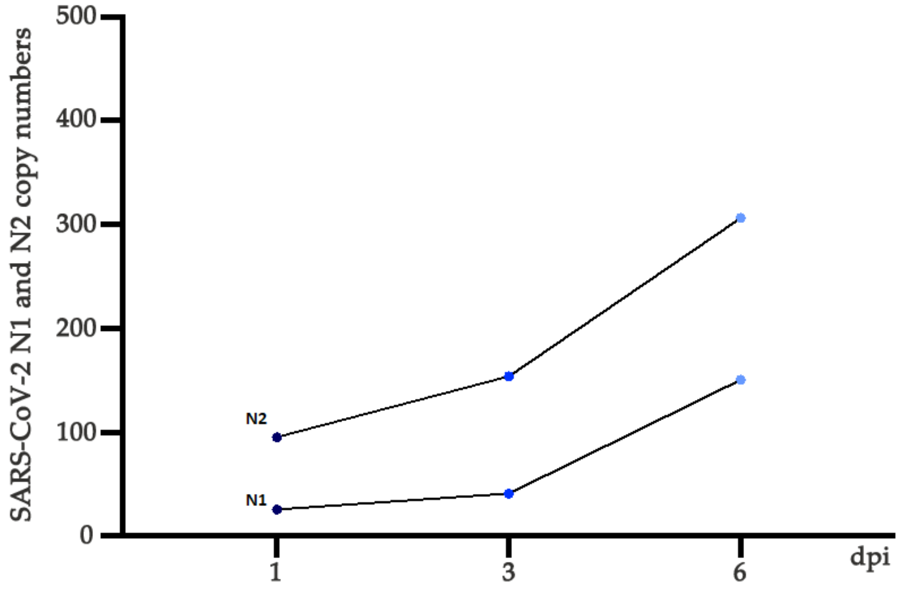

Figure S1. Replication of SARS-CoV-2 in cultured cells. N1 and N2 are the nucleocapsid gene used as probe. Viral copies show a significant increase during the days after incubation.

Table S1. Inspected DEGs involved in stress with fold change < 0.5

Gene	HCN2-CTR Expression	HCN2-SARS-CoV-2 Expression	Fold Change	*q*-Value	Biological Process
AGO1	443.21	586.23	0.40	2.12×10^{-9}	Oxidative Stress Induced Senescence
AGO3	332.01	278.74	−0.25	7.26×10^{-3}	Oxidative Stress Induced Senescence
CDK4	1953.66	1751.20	−0.16	1.72×10^{-5}	Oxidative Stress Induced Senescence
CDK6	202.41	146.25	−0.47	1.49×10^{-4}	Oxidative Stress Induced Senescence
MAP4K4	1984.86	2168.69	0.13	2.28×10^{-4}	Oxidative Stress Induced Senescence
MAPKAPK2	1332.04	1604.95	0.27	1.14×10^{-11}	Oxidative Stress Induced Senescence
MAPKAPK3	214.41	254.99	0.25	1.82×10^{-2}	Oxidative Stress Induced Senescence
MDM2	1246.44	1154.97	−0.11	2.03×10^{-2}	Oxidative Stress Induced Senescence
MDM4	225.61	162.50	−0.47	4.93×10^{-5}	Oxidative Stress Induced Senescence
MINK1	943.23	792.48	−0.25	2.89×10^{-6}	Oxidative Stress Induced Senescence
RPS27A	5162.55	4139.88	−0.32	7.98×10^{-47}	Oxidative Stress Induced Senescence
TNRC6A	1160.83	877.48	−0.4	9.14×10^{-17}	Oxidative Stress Induced Senescence
TNRC6C	242.41	201.24	−0.27	1.56×10^{-2}	Oxidative Stress Induced Senescence
TXN	2690.48	2008.69	−0.42	7.36×10^{-41}	Oxidative Stress

					Induced Senescence
UBA52	5232.95	4827.36	−0.12	1.15×10^{-7}	Oxidative Stress Induced Senescence
UBB	16001.25	16868.27	0.08	1.68×10^{-10}	Oxidative Stress Induced Senescence
UBC	50524.63	55289.68	0.13	4.38×10^{-92}	Oxidative Stress Induced Senescence
ATM	2213 .66	2393.68	0.11	6.59×10^{-4}	DNA Damage/Telomere Stress Induced Senescence
CABIN1	1165.63	996.22	−0.23	2.37×10^{-6}	DNA Damage/Telomere Stress Induced Senescence
CDK2	174.40	126.25	−0.47	4.99×10^{-4}	DNA Damage/Telomere Stress Induced Senescence
CDKN1B	200.01	263.74	0.40	1.10×10^{-4}	DNA Damage/Telomere Stress Induced Senescence
EP400	1323.24	963.72	−0.46	1.45×10^{-23}	DNA Damage/Telomere Stress Induced Senescence
KAT5	410.41	348.74	−0.23	5.15×10^{-3}	DNA Damage/Telomere Stress Induced Senescence
MRE11	288.81	393.74	0.45	8.07×10^{-8}	DNA Damage/Telomere Stress Induced Senescence
SOD1	4268.92	3819.89	−0.16	3.65×10^{-11}	Oxidative Stress alteration

The fold change columns are based on $\log_2$(HCN2-CTR expression / HCN2-SARS-CoV-2 expression). The values are rounded to the second decimal digit.
